# Protective Effect and Mechanisms of Eckol on Chronic Ulcerative Colitis Induced by Dextran Sulfate Sodium in Mice

**DOI:** 10.3390/md21070376

**Published:** 2023-06-25

**Authors:** Mengfan Liao, Songyi Wei, Xianmin Hu, Juan Liu, Jun Wang

**Affiliations:** Hubei Province Key Laboratory of Occupational Hazard Identification and Control, Institute of Pharmaceutical Innovation, School of Medicine, Wuhan University of Science and Technology, Wuhan 430065, China

**Keywords:** Eckol, chronic ulcerative colitis, inflammation, apoptosis, immunoregulation, TLR4 pathway

## Abstract

The use of functional foods and their bioactive components is receiving increasing attention as a complementary and alternative therapy for chronic ulcerative colitis (UC). This study explored the protective effect and mechanisms of Eckol, a seaweed-derived bioactive phlorotannin, on the dextran sodium sulfate (DSS)-induced chronic UC in mice. Eckol (0.5–1.0 mg/kg) reduced DSS-enhanced disease activity indexes, and alleviated the shortening of colon length and colonic tissue damage in chronic UC mice. The contents of tumor necrosis factor (TNF)-α, interleukin (IL)-1β, and IL-6 were significantly decreased, and the level of anti-inflammatory IL-10 was enhanced in the serum and colonic tissues collected from Eckol-treated mice compared with the DSS controls. Eckol administration significantly reduced the number of apoptotic cells and the expression of cleaved Caspase-3, and increased the B-cell lymphoma-2 (Bcl-2)/B-cell lymphoma-2- associated X (Bax) ratio in DSS-challenged colons. There were more cluster of differentiation (CD)11c^+^ dendritic cells and CD8^+^ T cells, and less CD4^+^ T cells infiltrated to inflamed colonic tissues in the Eckol-treated groups. Expression of colonic Toll-like receptor 4 (TLR4), nuclear factor kappa-B (NF-κB) p65, phosphorylated-signal transducer and activator of transcription (pSTAT)3 was significantly down-regulated by Eckol compared with the DSS-challenged group. In conclusion, our data suggest that Eckol appeared to be a potential functional food ingredient for protection against chronic UC. The anti-colitis mechanisms of Eckol might be attributed to the down-regulation of the TLR4/NF-κB/STAT3 pathway, inhibition of inflammation and apoptosis, as well as its immunoregulatory activity.

## 1. Introduction

As one of the common sub-types of inflammatory bowel diseases, ulcerative colitis (UC) has pathological characteristics of idiopathic, chronic, recurrent, and non-specific inflammatory lesions in the colonic mucosa [1,2]. Primary clinical features of UC are diarrhea, abdominal pain, and bloody mucus, which constantly fluctuate with alternating phases of exacerbation and remission, thus reducing health-related quality of life [1,2]. Long-term uncontrolled inflammation occurring in the colons of chronic UC patients could lead to irreversible intestinal damage, increased risk of colorectal cancer, and even death [2,3]. Despite the uncertain pathogenesis of UC, the interaction of multiple factors including genetic susceptibility, intestinal barrier dysfunction, immune imbalance, dietary, and environmental factors, etc., has been considered to be involved in its development and progression [2,4]. Currently, conventional agents such as mesalazine, corticosteroids, and immunomodulators have efficacy in achieving clinical remission for patients with active UC, but are associated with considerable serious adverse effects [2,5,6]. In recent years, the introduction of appropriate nutritional intervention and functional foods has been accepted as a key element of therapy, especially for UC patients with clinical remission [5,6,7,8]. The Mediterranean diet based on a high intake of biologically active foods has shown promising results in therapeutic efficacy, and is thus recommended as an optional approach for UC patients to maintain remission [6]. In particular, food- or herb-derived anti-inflammatory ingredients such as curcumin have been clinically demonstrated to effectively alleviate disease symptoms and reduce the risk of recurrent exacerbation in supporting the treatment of chronic UC [6].

As a bioactive phlorotannin mainly isolated from marine brown algae, Eckol is a closed-chain trimer of phloroglucinol that has been known to have multiple health benefits, such as anti-oxidant [9,10,11,12], anti-inflammatory [12,13,14,15,16,17,18], anti-allergic [19], antibacterial [20,21], and anti-cancer [22,23,24] properties. In particular, the beneficial role of Eckol in inflammation-related conditions has been demonstrated in vitro and in vivo, suggesting its therapeutic potential for inflammatory disorders [12,13,14,15,16,17,18]. Eckol protected the human vein endothelial cells and mice against pro-inflammatory responses activated by high mobility group box 1 protein, the latter of which is a late acting inflammatory mediator of lipopolysaccharide (LPS)-induced or sepsis-induced mouse lethality [13]. The production and release of classical pro-inflammatory cytokines, including tumor necrosis factor (TNF)-α, interleukin (IL)-1, IL-4, IL-5, IL-6, etc., have been reported to be significantly suppressed by Eckol administration in LPS-stimulated human hepatoma HepG2 cells [14], in human HaCaT keratinocytes stimulated with interferon-gamma (IFN-γ) and TNF-α mixture [15] or *Propionibacterium acnes* [16], as well as in the liver tissues of mice treated with carbon tetrachloride [12]. Transdermally and orally administered Eckol inhibited mouse ear swelling caused by skin sensitizers, and the mechanism was apparently associated with suppression upon the release of pro-inflammatory mediator prostaglandin E_2_, and down-regulation of the cyclooxygenase-2 expression and activity [17,18].

Considering the chronic inflammatory nature of UC, we assumed that Eckol as an anti-inflammatory marine-derived functional dietary supplement might be a promising alternative therapy for chronic UC. To date, no prior study has been conducted to assess the potential of Eckol to control the chronic UC. Therefore, this study aimed to investigate the possible protective effect and mechanisms of the oral administration of Eckol on an experimental chronic UC animal model established by repeated dextran sulfate sodium (DSS) administration. 

## 2. Results and Discussion

### 2.1. Eckol Ameliorated Chronic UC Induced by DSS in Mice

**Figure 1:**
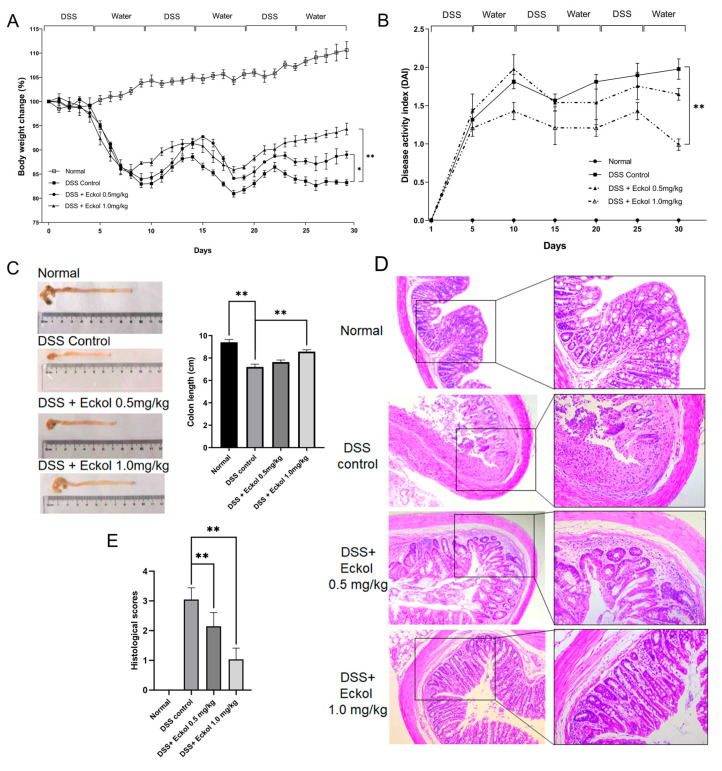
Effect of Eckol on body weight (**A**), DAI scores (**B**), colon length (**C**), representative H&E-stained colon sections (**D**), and histological scores (**E**) in DSS-induced chronic UC mice. Normal: the healthy mice; DSS control: DSS-induced chronic UC mice; DSS + Eckol 0.5 mg/kg: mice treated with DSS plus low-dose of Eckol; DSS + Eckol 1.0 mg/kg: mice treated with DSS plus high-dose of Eckol. Results are expressed as the mean ± SEM (*n* = 10 per group); * *p* < 0.05, ** *p* < 0.01.

The protective potential of Eckol treatment against chronic UC was evaluated by monitoring body weight loss, scoring the disease activity index (DAI), measuring colon length, and colonic histopathological analysis. As shown in Figure 1A, after three cycles of DSS administration, the body weights of mice in the chronic UC model group remarkably decreased compared with those of the normal mice, but the chronic UC mice with Eckol treatment restored their body weights (*p* < 0.05; *p* < 0.01). DAI scoring was performed to simultaneously assess the main clinical symptoms of UC, including weight loss, diarrhea, and hematochezia [25]. According to the monitoring of DAI scores, the normal control mice had a DAI score of 0 over the entire period of the experiment, and the mice chronically receiving DSS-supplemented water exhibited a gradual increase in DAI scores (Figure 1B). Importantly, compared with the chronic UC model group, the high-dose Eckol treatment markedly decreased the DAI scores in mice (*p* < 0.01), suggesting Eckol also effectively improved diarrhea and hematochezia. As shown in Figure 1C, at the end of the experiment, the colon length in the DSS-induced chronic UC mice was significantly shortened by 25% compared with the normal mice (*p* < 0.01); however, Eckol treatment at a dose of 1.0 mg/kg improved colorectal shortening in chronic UC mice by 14.3% (*p* < 0.01). Hematoxylin and eosin (H&E) staining of colonic tissues (Figure 1D) revealed that the three cycles of DSS administration caused severe mucosal and submucosal damage, characterized by disruption of the epithelial integration and crypt structure, as well as inflammatory cell infiltration, in the colonic tissues. The Eckol-treated mice displayed relatively intact colonic architectures, less infiltration of inflammatory cells, and improved colonic mucosal damage compared with the DSS controls. Then, a histological grading system ranging from 0 (normal) to 3 (severe and transmural inflammation, goblet cell loss >50%, and crypt density decrease by >50%) [25] was used for the quantification of colonic tissue damage. The results showed that the histological scores in the Eckol treatment groups were significantly decreased compared with those of the DSS control group (Figure 1E, *p* < 0.01), suggesting that Eckol treatment effectively improved the DSS-induced histopathological injuries in colonic tissues.

As a chronic inflammatory bowel disease, UC has become a major challenge in clinical treatment due to its unclear pathogenesis, high risk of recurrence, and poor prognosis [2]. Although there are multiple treatment options available to UC patients, there is still an unmet need for discovering and developing novel agents with more efficacy and safety [2,5]. In the past few years, based on the fact that dietary intervention has shown promising results in regulating intestinal inflammation and improving symptoms, the complementary and alternative medicines derived from natural sources are gaining more and more attention in the control of UC as dietary additives or health products [5,6,7,8]. In particular, as a marine-derived food traditionally consumed in East Asia, seaweed has been paid special attention as the basis for dietary supplements, functional food products, and drugs for the prevention and treatment of UC [5]. Some biologically active substances, for example, polysaccharides, isolated from seaweed have been proven to exert anti-inflammatory and gastroprotective effects [5]. Using the chronic experimental colitis model in mice induced by repeated DSS challenges, a simple, repeatable, and controllable animal model having similar clinical symptoms and histological changes to human chronic UC [26], our study firstly revealed that the oral administration of Eckol, a phlorotannin from seaweed, showed a significant protective role against the development of chronic UC in mice, which was manifested by reducing the DSS-enhanced DAI score, alleviating colon shortening, and improving colonic tissue damage in Eckol-treated chronic UC mice. These data suggested that Eckol as a marine anti-colitic agent represents an effective alternative for chronic UC patients.

### 2.2. Eckol Regulated the Levels of Inflammation-Related Cytokines in Chronic UC Mice

We further examined the effect of Eckol treatment on the levels of classic pro-inflammatory cytokines (TNF-α, IL-1β, and IL-6), as well as anti-inflammatory cytokine IL-10 in the serum and colon tissue samples collected from DSS-induced chronic UC mice. As shown in Table 1, DSS administration significantly enhanced the serum and colonic levels of TNF-α, IL-1β, and IL-6, and inhibited IL-10 production compared with the normal control group (*p* < 0.05; *p* < 0.01). Importantly, Eckol treatment dramatically restrained the production of pro-inflammatory cytokines and increased the IL-10 secretion levels in serum and colon tissues of DSS-administrated mice (*p* < 0.05; *p* < 0.01), suggesting that Eckol could effectively mitigate the systemic and local inflammation of colons in chronic UC mice.

The increased pro-inflammatory milieu and reduced anti-inflammatory response are significantly correlated with the occurrence and severity of intestinal mucositis during UC development [27]. As the prototypical inflammatory cytokines produced by various cells in the intestinal mucosa, TNF-α, IL-1β, and IL-6 induce pro-inflammatory effects in inflamed mucosal tissues, and have been verified to play a dominant and catalytic role in the progression of UC, especially during the early-onset phase [28]. As a loss-of-function mutation in a potent anti-inflammatory cytokine, IL-10 has been found to be strongly associated with severe inflammatory bowel disease [29]. In fact, the anti-inflammatory agents that suppress the production of pro-inflammatory mediators and increase anti-inflammatory response have long been considered as the cornerstone of symptomatic relief for UC patients [30]. In accordance with previous findings showing the pro-inflammatory property of Eckol [12,14], we found that Eckol significantly restrained the production of TNF-α, IL-1β, and IL-6 in the serum and colon tissues of chronic UC mice. Furthermore, the DSS-suppressed systemic and local levels of anti-inflammatory IL-10 were remarkably up-regulated by Eckol, which was consistent with a previous study [12] showing that Eckol promoted IL-10 expression in carbon tetrachloride-treated mouse liver tissues. These data confirmed the anti-inflammatory activity of Eckol under the condition of chronic UC.

### 2.3. Eckol Protected against DSS-Induced Colonic Cell Apoptosis 

**Figure 2:**
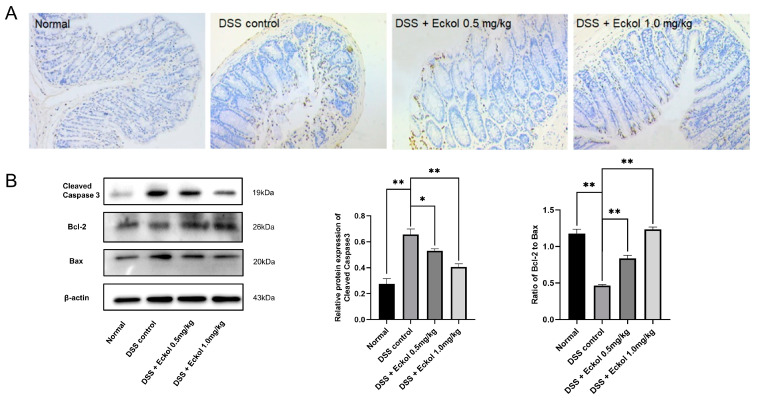
Effect of Eckol on the representative images of the TUNEL analysis for apoptotic cells (**A**) and the protein expression levels of cleaved-Caspase 3, Bcl-2, and Bax (**B**) in colon tissues of DSS-induced chronic UC mice. Normal: the healthy mice; DSS control: DSS-induced chronic UC mice; DSS + Eckol 0.5 mg/kg: mice treated with DSS plus a low-dose of Eckol; DSS + Eckol 1.0 mg/kg: mice treated with DSS plus high-dose of Eckol. Results are expressed as the mean values ± SEM (*n* = 10 per group); * *p* < 0.05, ** *p* < 0.01.

During the development of chronic UC, the intestinal cell apoptosis caused by inflammatory stress directly contributes to mucosal barrier dysfunction and destruction of colon tissue integrity [31,32]. In the present study, the terminal deoxynucleotidyl transferase mediated dUTP nick end labelling (TUNEL) staining of colon sections was employed to detect colonic cell apoptosis, and the expression of apoptosis-related proteins including cleaved-Caspase 3, B-cell lymphoma-2-associated X (Bax), and B-cell lymphoma-2 (Bcl-2) was measured using Western bolt. As shown in Figure 2A, there were plenty of TUNEL-positive apoptotic cells in the mucosal layer of DSS-challenged colons, but the Eckol treatment resulted in a lower percentage of apoptotic cells compared with the DSS controls. Accordingly, Western bolt analysis confirmed that Eckol remarkably down-regulated the protein expression of pro-apoptotic cleaved-Caspase 3 and Bax, and simultaneously up-regulated the anti-apoptotic Bcl-2 expression in the colonic tissues of DSS-challenged mice (Figure 2B, *p* < 0.05; *p* < 0.01). The expression ratio of Bcl-2 to Bax decreased by DSS administration was dose-dependently reversed by Eckol treatment (*p* < 0.01). 

The Bcl-2 protein family, including pro-apoptotic Bax and anti-apoptotic Bcl-2, play vital roles in the mitochondrial-dependent apoptotic pathway, and the Bcl-2/Bax ratio is commonly employed to evaluate the colonic apoptosis during chronic UC [30]. Caspase-3 is a key enzyme that can cleave other protein substrates, thus activating various apoptosis stimulators, and activation of the Caspase-3 apoptotic cascade is involved in pathogenicity of chronic UC [3]. In this study, we demonstrated that Eckol, especially at a high dose, could significantly inhibit colonic cell apoptosis in chronic UC mice. This finding was consistent with considerable previous reports on the anti-apoptotic activity of Eckol in HaCaT keratinocytes [11], murine hepatocytes [12], intestinal stem cells [33], and lung fibroblast (V79-4) cells [34]. Therefore, the preventive effect of Eckol on chronic UC might be, at least partly, due to its anti-apoptotic activity.

### 2.4. Eckol Modulated the Infiltration of Immune Cells in DSS-Challenged Colonic Tissues

UC has long been recognized as an autoimmune disease, which may occur due to impaired immune tolerance mechanisms leading to the development of pro-inflammatory T cells and abnormal immune responses [35,36]. As the most important professional antigen-presenting cells contributing to the regulation of inflammation and immunity, dendritic cells (DCs) play a crucial role in intestinal immune homeostasis, especially in inducing and maintaining immune tolerance to prevent overt inflammatory and immune responses to the gut microbiota [35,37]. Eckol has been found to protect in vitro DCs against apoptosis [38], and to recruit DCs into the liver tissues in carbon tetrachloride-treated mice [12], suggesting its potential immunomodulatory role associated with DCs.

**Figure 3:**
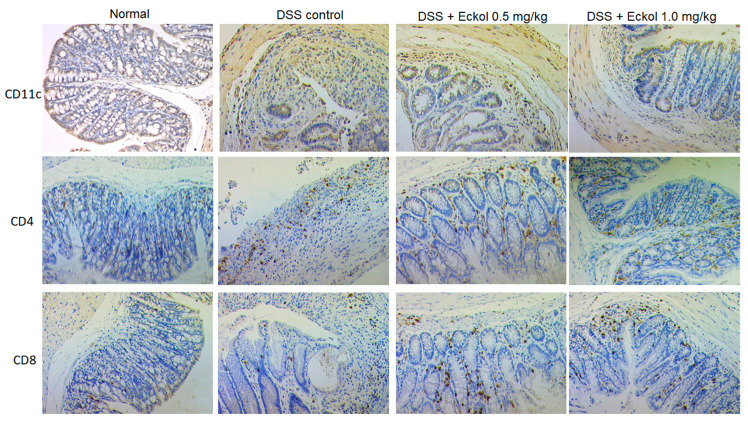
Representative images of immunohistochemical staining for CD11c^+^ DCs, CD4^+^, and CD8^+^ T cells in colon tissues of DSS-induced chronic UC mice. Normal: the healthy mice; DSS control: DSS-induced chronic UC mice; DSS + Eckol 0.5 mg/kg: mice treated with DSS plus low-dose of Eckol; DSS + Eckol 1.0 mg/kg: mice treated with DSS plus high-dose of Eckol.

To explore the effect of Eckol treatment on DC infiltration in the colon tissues of chronic UC mice, immunohistochemical detection for the cluster of differentiation (CD)11c, an accredited DC-specific marker, was performed. Representative images of immunohistochemical staining, shown in Figure 3, display that, more CD11c^+^ DCs in Eckol-treated groups were found to infiltrate into the mucosal, lamina propria, and submucosal areas of DSS-challenged colons. This finding suggests that Eckol induced DCs to migrate to the lesion sites. 

DCs infiltrating in colonic tissues could further affect the process of T lymphocyte activation and differentiation, thus regulating adaptive immune responses [35]. Activated T cells would differentiate into sub-populations of effector T cells, in particular, CD4^+^ and CD8^+^ T cells, then have an important role in the development of UC [35]. The effect of Eckol treatment on colonic tissue infiltration by T lymphocytes was detected by immunohistochemical staining for CD4 and CD8. As shown in Figure 3, a large number of CD4^+^ and CD8^+^ T lymphocytes aggregated in the DSS-challenged colons. Compared with the DSS model group, Eckol treatment decreased the number of CD4^+^ T cells, but increased the number of CD8^+^ T cells in the colonic tissues, suggesting that Eckol might contribute to inhibited CD4^+^ T-cell responses and enhanced CD8^+^ T-cell responses, resulting in an immunosuppressive micro-environment in chronic UC colons. Experimental evidence has shown that considerable ant-colitis agents or therapeutics reduced the ratio of CD4^+^ and CD8^+^ T lymphocytes in colonic tissues to maintain intestinal immune homeostasis, thereby helping to attenuate the progression of UC [39,40,41,42]. Similarly, our results demonstrate that Eckol increased CD11c^+^ DCs and meanwhile decreased the ratio of CD4^+^ to CD8^+^ T cells, which might indicate the regulatory effect of Eckol on intestinal immune tolerance.

### 2.5. Possible Involvement of Toll-like Receptor 4 (TLR4) Pathway

**Figure 4:**
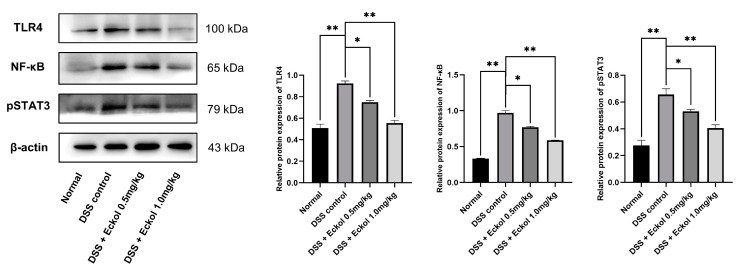
Effect of Eckol on the protein expression of TLR4, NF-κB, and pSTAT3 in colon tissues of DSS-induced chronic UC mice. Normal: the healthy mice; DSS control: DSS-induced chronic UC mice; DSS + Eckol 0.5 mg/kg: mice treated with DSS plus low-dose of Eckol; DSS + Eckol 1.0 mg/kg: mice treated with DSS plus high-dose of Eckol. Results are expressed as mean values ± SEM (*n* = 10 per group); * *p* < 0.05, ** *p* < 0.01.

In order to explore the possible molecular mechanisms underlying the anti-colitis effect of Eckol, the expression of TLR4 pathway-related proteins, including TLR4, nuclear factor kappa-B (NF-κB) p65, and phosphorylated signal transducer and activator of transcription (pSTAT)3 in the colonic tissues was measured by Western blot. As shown in Figure 4, Eckol treatment down-regulated the DSS-elevated colonic expression levels of TLR4, NF-κB p65, and pSTAT3 in chronic UC mice in a dose-dependent manner (*p* < 0.05; *p* < 0.01), suggesting an inhibitory activity of Eckol treatment on the TLR4/NF-κB/ STAT3 pathway in the colons of chronic UC mice.

Over-activation of the classical immune-inflammatory TLR4 signaling pathway within the colonic mucosa has a vital role in UC onset and progression [43,44]. Enhanced activation of TLR4, as a receptor participating in antigen recognition, has been found in the inflamed mucosa of UC patients [45]. TLR4 can further regulate the activation of downstream phosphorylated NF-κB (p65), the latter of which is also a key mediator of inflammation, innate immunity, and tissue integrity, via a signal transduction process. Therefore, the up-regulation of the colonic TLR4/NF-κB axis eventually leads to improper immune development, activation of the pro-apoptotic pathway and inflammatory cascade, colonic injury, and altered epithelial barrier, which are responsible for UC pathological features including neutrophil inflitration, distortion of the mucosal lining, edema, erosion, and ulceration [46,47]. Furthermore, NF-κB communicates with STAT3, another target molecule of the TLR4 signaling pathway, at multiple points and thereby accelerates inflammation and immune injury during UC development [25]. Zhang et al. [23] proved that Eckol could inhibit Reg3a-induced increases in the expression of STAT3 and NF-κB in the in vitro human pancreatic cancer cells. Lee and his colleagues reported that Eckol reduced the expression of NF-κB p65 in impaired PC12 cells induced by the amyloid-beta peptide (Aβ25-35) [48]. Furthermore, Eckol inhibited the IFN-γ/TNF-α- mediated nuclear translocation of NF-κB p65 in HaCaT keratinocytes [15]. This study firstly found that Eckol exerted a definite inhibitory effect on the DSS-upregulated TLR4/STAT3/ NF-κB signaling in colonic tissues at the molecular level, which might contribute to the anti-inflammatory and immunomodulatory roles of Eckol in chronic UC mice.

## 3. Materials and Methods

### 3.1. Animals and Chemicals

C57BL/6J specific pathogen-free (SPF) mice (female, 6–7 weeks old, 18–22 g) were obtained from the Laboratory Animal Center of Hubei Province (Hubei, China). The experimental protocols were approved by the Laboratory Animal Ethical Committee of Wuhan University of Science and Technology (approval number 2022239) in conformance to the ARRIVE guidelines, and was carried out according to the EU 2010/63/EU Animal Experiment Directive.

Eckol, obtained from Rongbao Environmental Technology Co., Ltd., Wuhan, China, was extracted and purified from brown alga *Ecklonia Cava* according to previously described protocol [33]. Briefly, the dried *Ecklonia Cava* was crashed then extracted with 80% methanol at room temperature for 24 h, and this extraction step was repeated three times. The extracted liquid was vacuum dried to obtain the crude extract from *Ecklonia Cava*. Then, the ethyl acetate fraction was partitioned from crude extract and further purified by HPLC. About 20 mg of Eckol was yielded from 1 kg dry weight of *Ecklonia Cava* after purification.

DSS (molecular weight: 36–50 kDa) was purchased from MP Biomedicals, Aurora, CO, USA.

### 3.2. Animal Model and Eckol Treatment

After a 1 week of adaptive feeding, the animals were randomly divided into four groups with ten mice in each group: normal, DSS control, Eckol low-dose (0.5 mg/kg), and high-dose (1.0 mg/kg) treatment groups. The doses of Eckol were chosen based on a previous study regarding the in vivo anti-inflammatory activity of Eckol in mice [12] and preliminary experiments. After pre-treatment with Eckol or the vehicle (distilled water) in all of the groups for 7 days, a chronic UC animal model was developed by administering three cycles of DSS to mice in the DSS control group and Eckol treatment groups. Each cycle consisted of DSS administration in drinking water at 2% for 5 days, followed by normal drinking water for 5 days. All of the mice were given free access to water. The daily water intake by mice in all groups was monitored, and no significant differences were found among groups. Animals in the Eckol treatment groups were orally given Eckol at the corresponding doses throughout the experimental period, and those in the normal and DSS control groups were gavaged with an equal volume of the vehicle. The weight of mice was recorded daily, and the weight loss was determined as the percentage change from initial body weight before DSS administration. A DAI score was calculated every five days in the mice according to a previously described method [25]. At 1 h after the final treatment of Eckol, the mice were anesthetized, the eyeballs were removed for blood collection, then sacrificed by cervical dislocation. 

### 3.3. Colon Length Measurement and Histopathological Analysis

After measuring the colon length with a ruler, the distal colon tissues were fixed with 4% paraformaldehyde, dehydrated and embedded in paraffin, then cut into tissue sections and stained with H&E for histopathological analysis. Histological scores of colonic tissues were estimated as previously described [25].

### 3.4. Measurement of Inflammation-Related Cytokine Levels in Serum and Colon Tissues

The blood samples were centrifuged to obtain the serum samples. The colonic tissues were weighed and then homogenized with phosphate buffered brine (PBS) (*w*/*v*, 1:9). Then, homogenate liquid was centrifuged at 4000 rpm for 10 min at 4 °C, and the supernatant was collected. The levels of TNF-α, IL-1β, IL-6, and IL-10 in the serum samples and colonic tissue homogenates were measured according to the enzyme-linked immunosorbent assay (ELISA) kit instructions (R&D Systems, Minneapolis, MN, USA). Protein contents in colon tissue homogenates were monitored by the bicinchoninic acid disodium assay kit (Nanjing Jiancheng Bioengineering Institute, Nanjing, China).

### 3.5. TUNEL Assay

Apoptotic cells in the colonic mucosa were detected using the TUNEL in situ apoptosis detection kit (Shanghai Kaifeng Biotechnology Development Co., Ltd., Shanghai, China). As described in the previous protocol [24], the sections were dewaxed, hydrated, and blocked with 3% hydrogen peroxide (H_2_O_2_) in methanol for 10 min. After being permeabilized with protease K at 37 °C for 25 min, the sections were then incubated with TUNEL reaction solution at 37 °C for 1 h. The converter-POD solution was added and incubated for 30 min in the dark, then stained with 3,3’-diaminobenzidine (DAB).

### 3.6. Immunohistochemical Staining 

As previously described [24,42], the paraffin-embedded colon tissue sections were hatched with the rabbit anti-CD11c, CD4, and CD8 antibodies (Servicebio, Wuhan, China), then a biotinylated goat anti-rabbit secondary antibody (Servicebio, Wuhan, China). DAB was used for chromogenic development, and the cell nuclei were re-stained with hematoxylin.

### 3.7. Western Blot Analysis

As previously described [23,24], the RIPA protein lysate (Shanghai Beyotime Biotechnology Co., Ltd., Shanghai, China) was employed to extract the total proteins from fresh colon tissue homogenates. The proteins were quantified with the bicinchoninic acid (BCA) protein assay kit (Biosharp, Wuhan, China) and denatured at 95 °C for 10 min, then separated by 12% SDS-PAGE gel electrophoresis, and transferred to the nitrocellulose membrane by electroblotting. The membrane was blocked with 5% skim milk, then incubated with primary antibodies against TLR4 (Santa Cruz Biotechnology, Santa Cruz, CA, USA), and cleaved Caspase 3, Bcl-2, Bax, NF-κB p65, p-STAT3 (Cell Signaling Technology, Danvers, MA, USA), and β-actin (Proteintech, Wuhan, China) overnight at 4 °C. The HRP-labeled goat anti-rabbit or mouse secondary antibodies (Servicebio, Wuhan, China) were added to incubate for 1 h. The gray value of each strip was measured after ECL (Wuhan Kerui Biotechnology Co., Ltd., Wuhan, China) luminous development. β-actin was utilized as the control in the same sample.

### 3.8. Statistical Analysis

Data were expressed as the mean ± SEM. SPSS 25.0 software was applied for homogeneity test of variance and one-way analysis of variance (ANOVA) to analyze the differences between groups. Post hoc Tukey HSD test was used for repeated measure analysis of variance to analyze changes in body weight and DAI score among groups. The *p* < 0.05 or *p* < 0.01 was regarded as statistically significant.

## 4. Conclusions

**Figure 5:**
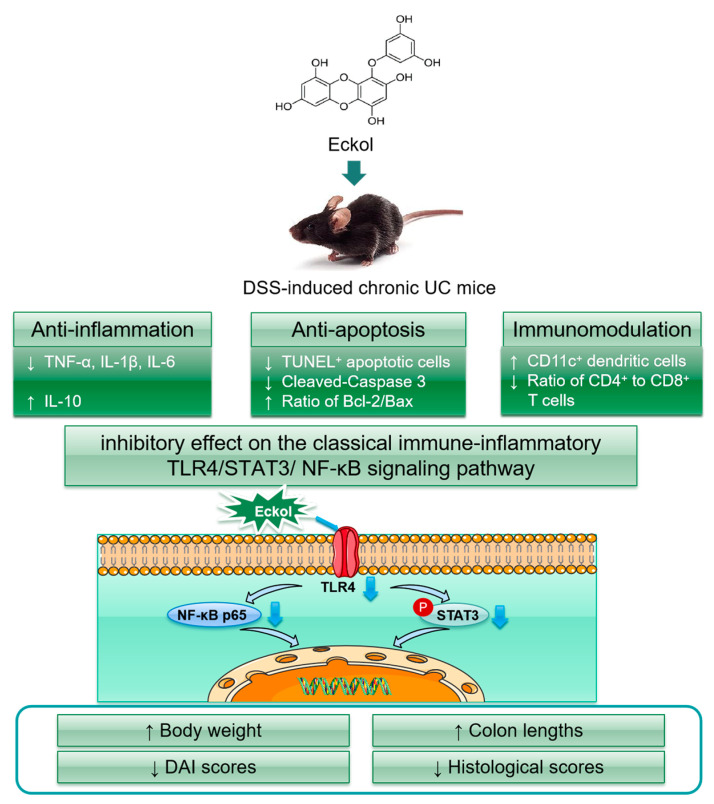
Mechanisms of action of Eckol in DSS-induced chronic UC mice. Bax, B-cell lymphoma-2-associated X; Bcl-2, B-cell lymphoma-2; CD, cluster of differentiation; DAI, disease activity index; DSS, dextran sodium sulfate; IL, interleukin; NF-κB, nuclear factor kappa-B; STAT3, signal transducer and activator of transcription; TLR, Toll-like receptor; TNF, tumor necrosis factor; TUNEL, terminal deoxynucleotidyl transferase mediated dUTP nick end labelling; UC, ulcerative colitis.

Our study confirmed for the first time that the oral administration of Eckol inhibited the progression of chronic UC by alleviating colonic inflammation, protecting against apoptosis, recruiting CD11c^+^ DCs to inflamed colonic tissues, and inducing local immune tolerance. The molecular mechanism of Eckol against chronic UC in mice might be associated with its inhibitory effect on the classical immune-inflammatory TLR4/STAT3/ NF-κB signaling pathway (Figure 5). The above findings might provide support for the potential of Eckol as a dietary supplement or health product in the control of chronic UC.

## Figures and Tables

**Table 1:** Effects of Eckol on the levels of inflammation-related cytokines in the serum and colon tissue samples collected from DSS-induced chronic UC mice (mean ± SEM, *n* = 10 per group).

Group	Dosage (mg/kg)	Serum Samples (pg/mL)				Colon Tissue Samples (pg/mg prot)			
		TNF-α	IL-1β	IL-6	IL-10	TNF-α	IL-1β	IL-6	IL-10
Normal	-	63.4 ± 2.9	104.6 ± 6.8	84.1 ± 5.0	110.7 ± 10.5	8.0 ± 1.9	91.6 ± 6.7	69.3 ± 5.3	43.5 ± 6.5
DSS control	-	212.5 ± 23.3 **	435.8 ± 24.2 **	397.1 ± 16.7 **	97.3 ± 12.3 *	49.3 ± 8.8 **	488.5 ± 18.4 **	318.0 ± 17.3 **	18.1 ± 9.5 **
Eckol	0.5	137.0 ± 19.5 ^# #^	309.1 ± 25.7 ^# #^	218.3 ± 30.8 ^# #^	106.1 ± 13.5	34.2 ± 8.9 ^# #^	330.5 ± 18.8 ^# #^	201.1 ± 20.7 ^# #^	29.4 ± 8.8 ^# #^
	1.0	131.2 ± 22.0 ^# #^	243.3 ± 28.2 ^# #^	207.6 ± 24.1^# #^	109.4 ± 11.8 ^#^	27.5 ± 8.4 ^# #^	286.8 ± 20.3 ^# #^	168.5 ± 17.6 ^# #^	32.9 ± 7.6 ^# #^

* *p* < 0.05, ** *p* < 0.01, vs. the normal group; ^#^ *p* < 0.05, ^# #^ *p* < 0.01, vs. the DSS control group.

## Data Availability

The data presented in this study are available on request from the corresponding author.
